# Plasma SH Concentrations and Mortality in Patients with Newly Diagnosed Idiopathic Pulmonary Fibrosis

**DOI:** 10.3390/antiox15080923

**Published:** 2026-07-24

**Authors:** Panagiotis Paliogiannis, Stefano Zoroddu, Simona Fois, Chiara Scala, Elisabetta Zinellu, Arduino A. Mangoni, Ciriaco Carru, Pietro Pirina, Angelo Zinellu, Alessandro G. Fois

**Affiliations:** 1 Department of Medicine, Surgery and Pharmacy, University of Sassari, Viale San Pietro 43, 07100 Sassari, Italy; pirina@uniss.it (P.P.); agfois@uniss.it (A.G.F.); 2 Department of Biomedical Sciences, University of Sassari, Viale San Pietro 43, 07100 Sassari, Italy; szoroddu@uniss.it (S.Z.); carru@uniss.it (C.C.); azinellu@uniss.it (A.Z.); 3 Unit of Pulmonology and Respiratory Diseases, University Hospital (AOU) of Sassari, Viale San Pietro 43, 07100 Sassari, Italy; simonafois90@gmail.com (S.F.); chiara.scala.cs93@gmail.com (C.S.); elisabetta.zinellu@aousassari.it (E.Z.); 4 Discipline of Clinical Pharmacology, College of Medicine and Public Health, Flinders University, Sturt Road, Bedford Park, Adelaide, SA 5042, Australia; arduino.mangoni@flinders.edu.au; 5 Department of Clinical Pharmacology, Flinders Medical Centre, Southern Adelaide Local Health Network, Flinders Drive, Bedford Park, Adelaide, SA 5042, Australia

**Keywords:** idiopathic pulmonary fibrosis, oxidative stress biomarkers, PSH, TBARS, redox imbalance

## Abstract

Introduction. Oxidative stress plays a critical role in the pathogenesis of idiopathic pulmonary fibrosis (IPF), yet its prognostic significance remains unclear. This study investigated the association between plasma sulfhydryl (SH) group concentrations and thiobarbituric acid reactive substances (TBARS), systemic markers of oxidative stress, and mortality in patients with IPF. Materials and methods. Eighty-eight patients with newly diagnosed IPF were recruited between 2016 and 2023 for the purposes of the study. Plasma SH and TBARS were measured at baseline under standardized conditions and normalized to plasma protein content. Survival analyses were performed using Kaplan–Meier curves and Cox regression models, adjusting for lung function parameters and IPF stage. Results. Patients with lower SH concentrations had significantly higher mortality (log-rank *p* = 0.012). SH group concentrations, but not TBARS, were independently and negatively associated with survival in multivariate models adjusting for %TLC, %FVC, %DLCO, and IPF stage (HR: 0.606, 95% CI: 0.443–0.830, *p* = 0.0018). Conclusions. Low plasma SH concentrations, reflecting systemic redox imbalance, are independently associated with increased mortality in newly diagnosed IPF. SH quantification represents a promising prognostic biomarker in IPF.

## 1. Introduction

Idiopathic Pulmonary Fibrosis (IPF) is a chronic condition predominantly affecting adults that is characterized by progressive fibrosis of unknown etiology limited to the lungs [1,2]. IPF is characterized histologically and radiologically by a usual interstitial pneumonia (UIP) pattern and clinically by dyspnea on exertion, dry cough, and progressive decline in lung function [3,4]. The natural history of IPF is marked by substantial heterogeneity, with some patients experiencing a slow but steady progression, others undergoing episodes of acute exacerbation, and a minority maintaining stable lung function for prolonged periods [4,5]. Despite the availability of antifibrotic therapies such as pirfenidone and nintedanib, which can slow the rate of functional decline, the median survival remains short, approximately 3 to 5 years after diagnosis. At the same time, the observed heterogeneity in disease progression warrants the identification of robust prognostic biomarkers to inform clinical decision making and monitoring [6,7,8].

A growing body of evidence identifies oxidative stress as a critical contributor to the pathogenesis and progression of IPF [9]. Oxidative stress results from an imbalance between the production of reactive oxygen species (ROS) and the antioxidant defense system, favoring oxidative reactions that irreversibly damage lipids, proteins, and nucleic acids [10,11]. In IPF, major contributors to ROS production include activated alveolar macrophages and neutrophils, mitochondrial dysfunction, and environmental factors such as cigarette smoke and pollutants [12,13]. Oxidative stress can amplify fibrogenesis by promoting epithelial cell injury, myofibroblast differentiation, and the production of extracellular matrix components [14,15].

Among systemic biomarkers of oxidative stress, the plasma concentration of sulfhydryl (–SH) groups, primarily derived from protein-bound cysteine residues and reduced glutathione, provides an integrative measure of redox buffering capacity [10,16]. SH groups are susceptible to oxidation by ROS, resulting in disulfide formation and a decrease in free thiol content [17]. Reduced plasma SH concentrations have been associated with various oxidative stress-related diseases, including cardiovascular disease, chronic kidney disease, and sepsis [18,19,20]. This reflects a broader trend in chronic disease research, in which accessible blood-based markers have been increasingly explored for their diagnostic and prognostic utility across different pathological settings [21,22]. In contrast, thiobarbituric acid reactive substances (TBARS) reflect the presence of lipid peroxidation byproducts, particularly malondialdehyde (MDA), and serve as indirect markers of membrane lipid damage due to oxidative injury [9,23].

Despite the established pathophysiological role of oxidative stress in IPF, few studies have assessed the prognostic relevance of circulating redox biomarkers [24,25]. Furthermore, the potential utility of plasma SH group concentration as a predictor of mortality in IPF remains largely unexplored. Clarifying this relationship could provide novel insights into disease mechanisms and identify clinically meaningful biomarkers that are readily measurable and potentially modifiable.

The present study was therefore designed to investigate whether plasma SH concentrations, measured under fasting conditions and normalized to plasma protein content, are independently associated with all-cause mortality in a well-characterized cohort of patients with newly diagnosed IPF. We also examined the association between TBARS levels and survival to assess the contribution of lipid peroxidation to disease outcomes. A comprehensive clinical and functional evaluation, including lung function parameters and disease staging, was incorporated into the analysis to control for confounding variables.

## 2. Materials and Methods

### 2.1. Study Population

Patients with newly diagnosed IPF were consecutively recruited at the Respiratory Unit of the University of Sassari between 2016 and 2023. The follow-up period was defined as the time from diagnosis to death or to the censoring date (July 2024) among surviving patients. Baseline was defined as the time of the first blood sample collection, performed during the diagnostic visit and prior to the initiation of antifibrotic therapy. At that time, all patients were treatment-naïve. After diagnosis, patients underwent a scheduled clinical visit at one month from diagnosis and then at six-month intervals. For deceased patients, date of death was obtained from clinical records when available, or, if necessary, through structured telephone interviews with close relatives. The study was approved by the Ethics Committee of the University Hospital of Cagliari (Approval No. 2262/CE-2015/11/17), and written informed consent was obtained from all participants. The study was conducted in accordance with the principles of the Declaration of Helsinki. The diagnosis of IPF was made according to established clinical guidelines [26]. High-resolution computed tomography (HRCT) images and lung biopsy specimens were independently assessed by two experienced radiologists and two expert pathologists. Each case was subsequently discussed in a multidisciplinary meeting that included pulmonologists, radiologists, and pathologists specializing in interstitial lung diseases. The diagnosis was confirmed by surgical lung biopsy in nine of the enrolled patients.

### 2.2. Imaging and Pulmonary Function

Pulmonary function tests were performed for all patients and included forced expiratory volume in 1 s (FEV1), forced vital capacity (FVC), total lung capacity (TLC), and diffusing capacity of the lungs for carbon monoxide (DLCO). Disease stage was defined according to the GAP index (Gender, Age, Physiology), which combines age, sex, %FVC, and %DLCO to classify patients into stages I, II, and III of IPF severity. The results were expressed as percentages of the predicted values (%FEV1, %FVC, and %DLCO) following the standards set by the American Thoracic Society and the European Respiratory Society [26]. Patients experiencing acute IPF exacerbations or presenting with malignancy, bleeding disorders, or significant hepatic or renal dysfunction were excluded from the study.

### 2.3. PSH and TBARS Evaluation

Blood samples were drawn into EDTA tubes following an overnight fast and centrifuged at 4 °C at 1500× *g* for 10 min to separate plasma, which was then stored at −80 °C until further analysis. Plasma SH groups were quantified spectrophotometrically using 5,5′-dithiobis-2-nitrobenzoic acid (DTNB) as a chromogenic agent, with the absorbance of the resulting conjugate measured at 405 nm [27]. Sample concentrations were calculated using a GSH standard curve, and plasma SH concentrations were normalized to total plasma protein content, which was determined by the Lowry method.

TBARS concentrations were assessed according to Esterbauer and Cheeseman [28]. This method quantifies MDA and other aldehydic compounds formed as a result of lipid peroxidation induced by hydroxyl radicals. Plasma samples were treated with 10% trichloroacetic acid and 0.67% thiobarbituric acid, then incubated at 95 °C in a thermoblock for 30 min. A standard curve was generated using MDA, and TBARS concentrations were calculated based on the absorbance at 535 nm.

### 2.4. Statistical Analysis

The Kolmogorov–Smirnov test was employed to evaluate the distribution of variables. Data are presented as mean ± standard deviation (SD) or median with interquartile range (IQR), as appropriate. Group comparisons for continuous variables were performed using either the unpaired Student’s *t*-test or the Mann–Whitney U test, depending on the normality of the distribution. To assess the association between plasma SH concentrations and disease severity, correlation analyses were conducted using Pearson’s r for variables with normal distribution and Spearman’s rho for non-normally distributed variables. For survival analysis, the time of IPF diagnosis was defined as time zero, and survival probabilities were estimated using Kaplan–Meier curves, with death as the event of interest. Plasma SH and TBARS concentrations were categorized into tertiles to better illustrate the relationship with mortality.

Cox proportional hazards regression was used for both univariate and multivariate analyses. Categorical variables (e.g., smoking status and antifibrotic therapy) were entered into the Cox models according to the original three-level coding used in the dataset, with the reference category automatically defined by the software. The independent association between PSH and TBARS with survival was assessed using a multivariate Cox model, adjusted for potential confounders (stage, %FVC, %DLCO, and %TLC) identified through univariate analysis with a *p*-value < 0.05. To address the issue of multicollinearity, particularly between the GAP stage and its constituent variables (%FVC and %DLCO), we evaluated the prognostic value of PSH and TBARS using two separate models. Model 1 was adjusted for %TLC and the composite GAP stage, while Model 2 was adjusted for the individual physiological parameters: %TLC, %DLCO, and %FVC. Multicollinearity was assessed using the Variance Inflation Factor (VIF). All VIF values were below 3 in both models, indicating no substantial collinearity issues. The proportional hazards assumption was assessed using Schoenfeld residuals and the corresponding global test. No statistically significant violations of the proportional hazards assumption were detected for any of the Cox regression models.

All statistical analyses were performed using MedCalc for Windows, version 23.1.3 64-bit (MedCalc Software, Ostend, Belgium).

## 3. Results

A total of 88 IPF patients (67 men and 21 women) were included in the study (Table 1). Thirty-eight patients (43%) died during the study, while 50 (57%) survived. The mean follow-up duration was 51.9 ± 27.5 months for the entire patient cohort and differed significantly between survivors and non-survivors (61.8 ± 25.9 vs. 38.8 ± 24.2 months, *p* < 0.001).

The mean age at the time of diagnosis was 69.7 ± 7.4 years in the whole study population. The mean age of survivors and non-survivors was not significantly different (*p* = 0.95). There were no significant between-group differences in body mass index (BMI) or pre-existing diseases (diabetes, arterial hypertension, cerebrovascular diseases and atrial arrhythmias). In the survivor group, 58% of patients were in stage I, 32% in stage II, and 10% in stage III, whereas in the non-survivor group, 34% of patients were in stage I, 55% in stage II, and 11% in stage III, showing a trend toward significance (*p* = 0.07). Four patients were not taking antifibrotic medications, while 40 were taking nintedanib, and 44 were taking pirfenidone. No significant differences in drug utilization were observed between the two groups (*p* = 0.40).

**Figure 1:**
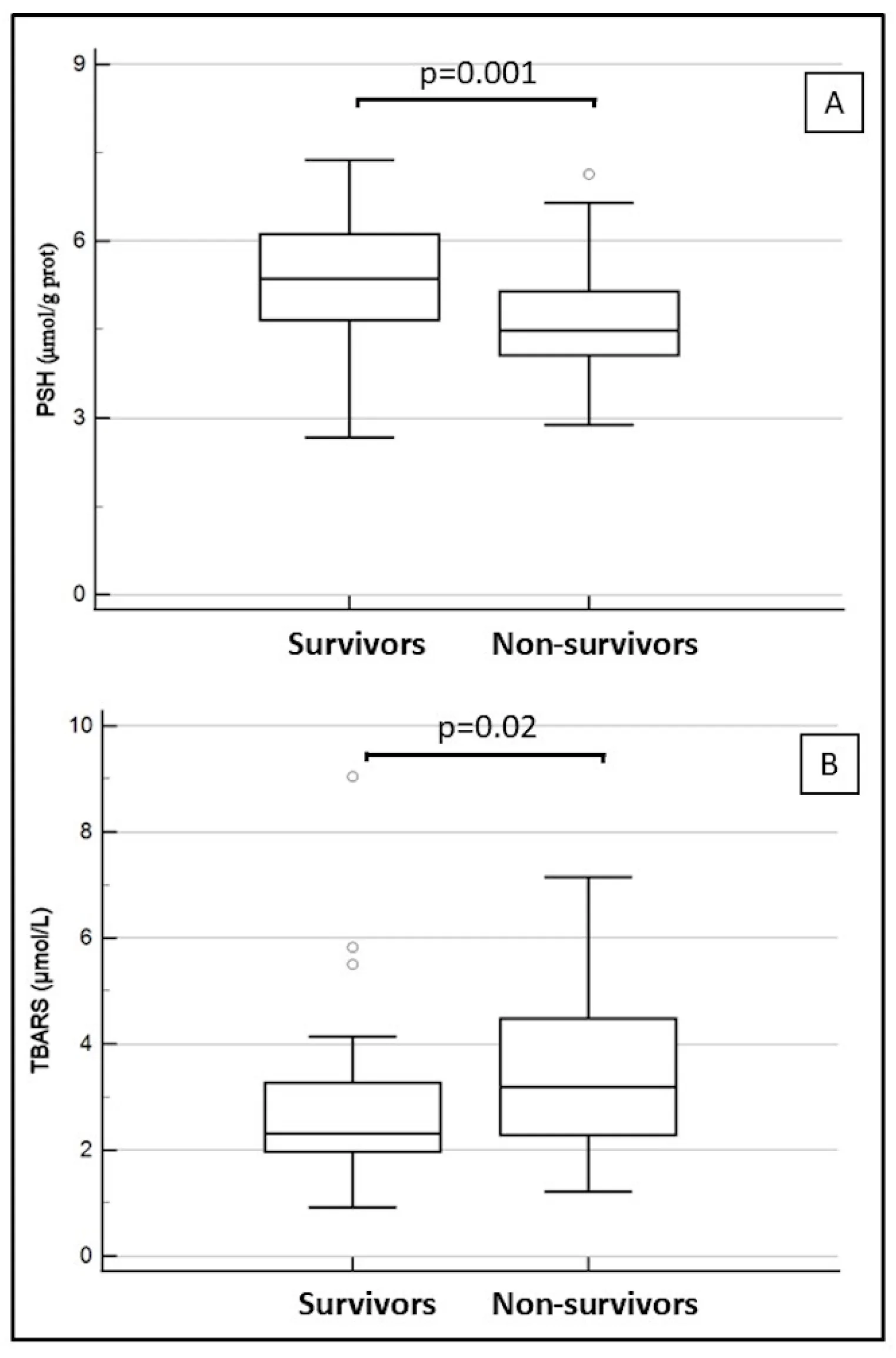
Comparison of oxidative stress markers between survivors and non-survivors with IPF. (**A**) PSH group concentrations were significantly lower in non-survivors compared to survivors (*p* = 0.001). (**B**) TBARS concentrations were significantly higher in non-survivors (*p* = 0.02). Data are presented as box-and-whisker plots showing median, interquartile range, and full data range (excluding outliers, which are shown as open circles).

A significant reduction in %TLC [median 69.8 (IQR 57.1–81.4) vs. 80.3 (IQR 68.1–88.8), *p* = 0.022] and %FVC (71.9 ± 18.6 vs. 80.4 ± 20.6, *p* = 0.047) was observed in non-survivors. In contrast, no significant between-group differences were observed in %FEV_1_ (*p* = 0.19) or %DLCO (*p* = 0.11). A significant decrease in 6MWT distance was also observed in the non-survivor group (302 ± 108 vs. 372 ± 160 m, *p* = 0.046). As shown in Figure 1, the non-survivor group exhibited significantly lower PSH concentrations, 4.64 ± 0.98 µmol/g prot vs. 5.42 ± 1.03 µmol/g prot, *p* = 0.001, and higher TBARS concentrations compared to the survivor group: median 3.19 (IQR 2.28–4.48) μmol/L vs. 2.31 (IQR 1.97–3.27) μmol/L, *p* = 0.02.

To evaluate the association between plasma SH concentrations and disease severity, correlation analyses were performed. PSH concentrations were not significantly correlated with %FVC (r = 0.033, *p* = 0.75), %DLCO (rho = 0.00, *p* = 0.95), %TLC (rho = 0.16, *p* = 0.14), or GAP stage (r = −0.09, *p* = 0.39). Only a weak, borderline significant correlation was found with 6 min walk test distance (r = 0.20, *p* = 0.07).

**Figure 2:**
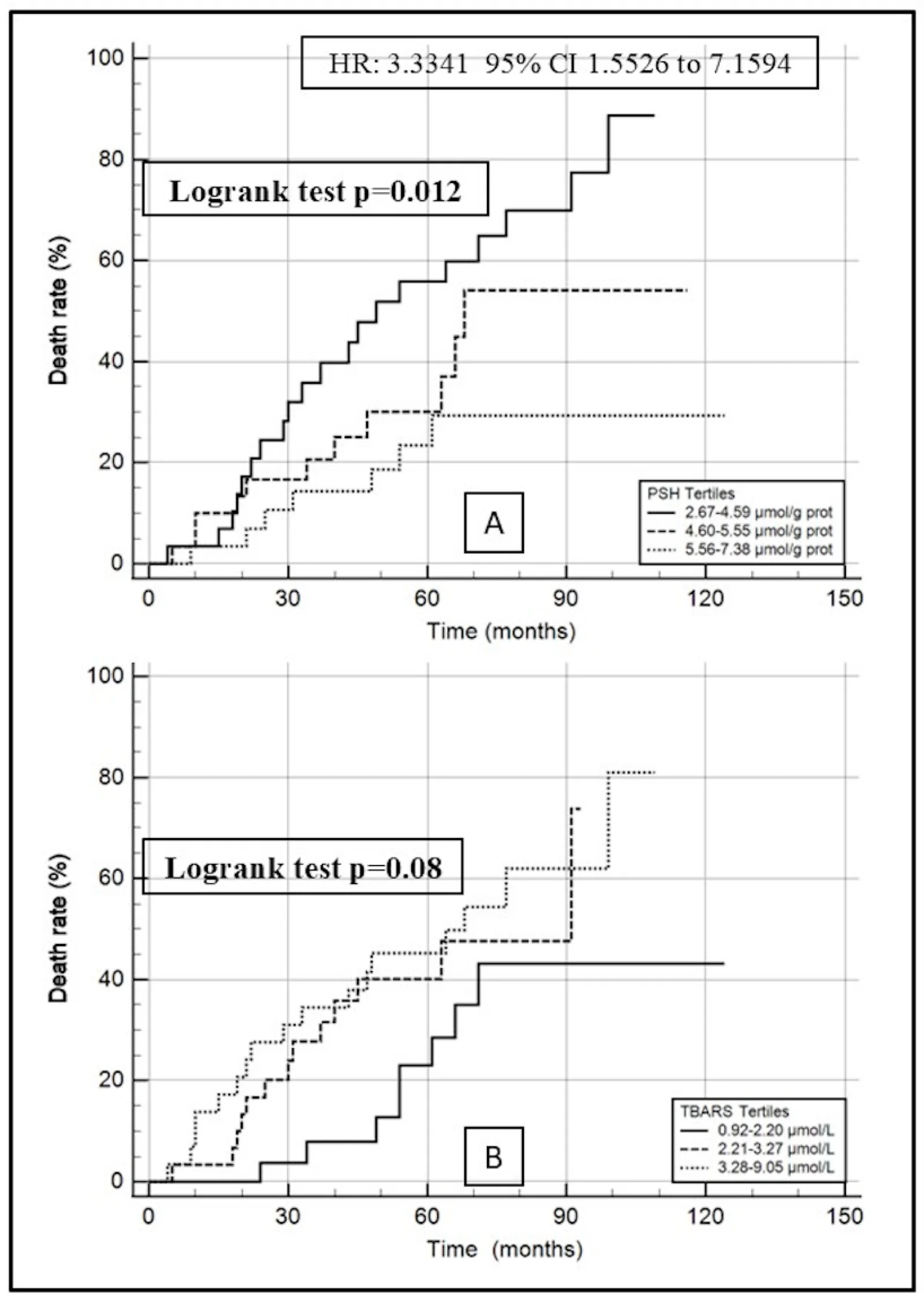
Kaplan–Meier survival curves according to plasma tertiles of oxidative stress markers in patients with IPF. (**A**) Patients in the lowest tertile of PSH group concentrations (≤4.59 µmol/g protein) exhibited significantly higher mortality compared to those in higher tertiles (log-rank test *p* = 0.012). The hazard ratio for mortality in the lowest vs. highest PSH tertile was 3.33 (95% CI: 1.55–7.16). (**B**) TBARS tertiles showed a non-significant trend toward increased mortality in patients with higher concentrations of lipid peroxidation (log-rank test *p* = 0.08). Survival time is expressed in months from diagnosis. Death rate is shown as cumulative percentage. Tick marks indicate censored observations.

Kaplan–Meier survival curves were used to evaluate mortality in IPF patients with different levels of plasma PSH and TBARS (Figure 2). A significant association between PSH and mortality was observed (log-rank test, *p* = 0.012; Figure 2A). Compared with patients with PSH in the third tertile (≥5.56 µmol/g prot), those in the first tertile (≤4.60 µmol/g prot) had a 3.33-fold higher risk of death (95% CI, 1.55–7.16). The mortality rate was 69% in the PSH tertile I (2.67–4.59 µmol/g prot), 37% in tertile II (4.60–5.55 µmol/g prot) and 24% in tertile III (5.56–7.38 µmol/g prot). A non-significant trend toward an association between TBARS and mortality was also observed (log-rank test, *p* = 0.08; Figure 2B).

Univariate Cox regression analysis, presented in Table 2, confirmed significant associations of PSH with survival (HR: 0.6567, 95% CI: 0.4915–0.8775, *p* = 0.0045) as well as a non-significant trend toward an association with TBARS (HR: 1.1737, 95% CI: 0.9855–1.3979, *p* = 0.0725). Additionally, survival showed significant associations with %TLC (HR: 0.9750, 95% CI: 0.9578–0.9926, *p* = 0.0054), %FVC (HR: 0.9803, 95% CI: 0.9630–0.9978, *p* = 0.0276), %DLCO (HR = 0.9807, 95% CI: 0.9623–0.9995, *p* = 0.045), and stage (HR: 1.6369, 95% CI: 1.0095–2.6541, *p* = 0.0457).

In multivariate Cox regression analysis (Table 3), the associations of PSH with survival remained significant even after adjusting for confounders in Model 1 (HR: 0.6637, 95% CI: 0.4979–0.8847, *p* = 0.0052) and Model 2 (HR: 0.6063, 95% CI: 0.4427–0.8302, *p* = 0.0018).

## 4. Discussion

In this study, lower plasma SH concentrations were independently associated with increased all-cause mortality in patients with newly diagnosed IPF, even after adjusting for established prognostic indicators such as %TLC, %FVC, %DLCO, and disease stage. Interestingly, unlike large epidemiological registries and clinical trials, age at diagnosis was not associated with mortality in our cohort. This may reflect the relatively homogeneous age distribution and limited sample size (88 patients and 38 events), which may have reduced the statistical power to detect broad demographic mortality predictors. The association between plasma SH concentrations and mortality remained robust across multivariate models, suggesting that circulating SH concentrations may represent a promising biomarker of poor outcome in IPF. Conversely, although TBARS concentrations were significantly elevated in non-survivors, their prognostic value did not reach statistical significance in multivariate analysis, indicating that lipid peroxidation alone may not capture the complexity of redox imbalance in this population.

Our findings contribute to a growing body of evidence implicating oxidative stress as a key driver of IPF progression [29]. Experimental models have shown that ROS mediate epithelial cell apoptosis, mitochondrial dysfunction, and the activation of profibrotic pathways, including TGF-β signaling and fibroblast–myofibroblast transition, all of which are fundamental to the fibrotic remodeling observed in IPF lungs [23,30,31,32]. While several studies have documented elevated oxidative stress markers in IPF lung tissue and bronchoalveolar lavage fluid, few have evaluated systemic redox parameters in relation to clinical outcomes [29,33]. The results of our study suggest that the systemic thiol pool, as reflected by plasma SH concentrations, may represent more than a surrogate marker of pulmonary oxidative burden and may provide insight into disease progression and severity [34,35].

Plasma SH groups, primarily derived from protein thiols and reduced glutathione, serve as crucial components of the endogenous antioxidant defense [36,37]. Their oxidation reflects not only the burden of oxidative stress but also a depletion of systemic antioxidant reserves [38,39]. In this regard, our results agree with studies in other chronic diseases, such as cardiovascular disease and chronic kidney disease, where reduced SH concentrations were predictive of mortality and adverse events [40,41]. Notably, the inverse association between SH concentrations and mortality in IPF mirrors similar findings in sepsis, where SH depletion correlates with multi-organ dysfunction and poor prognosis [42,43]. These parallels reinforce the notion that thiol redox status reflects systemic vulnerability to oxidative damage across diverse pathological states.

The observed reduction in plasma SH groups may contribute to IPF progression by impairing systemic antioxidant buffering capacity. Plasma thiols, primarily derived from protein-bound cysteines (especially albumin) and reduced glutathione, represent a major extracellular antioxidant defense system capable of scavenging ROS and preventing oxidative damage to lipids, proteins, and cell membranes. A decrease in circulating SH levels reflects a diminished redox reserve, which may exacerbate systemic oxidative stress and contribute to the progression of lung injury [43]. This systemic redox imbalance could promote IPF progression through several pathways. For instance, reduced thiol availability may exacerbate mitochondrial ROS production in alveolar epithelial cells and enhance NOX4 activity, a key source of oxidative stress in IPF that drives fibroblast activation and myofibroblast differentiation [24]. Furthermore, alterations in extracellular thiol redox status can influence redox-sensitive signaling, including TGF-β pathway activation, a central mediator of fibrogenesis [44]. Thus, low plasma SH concentrations may not only be a marker of oxidative stress but may also actively contribute to a pro-fibrotic environment by weakening antioxidant defenses and favoring oxidative modifications that sustain chronic inflammation and tissue remodeling.

The apparent superiority of SH groups over TBARS in predicting mortality may reflect the broader and more integrative nature of thiol-based redox regulation, which encompasses not only lipid peroxidation but also protein oxidation, redox signaling, and detoxification pathways [45,46]. TBARS, though widely used, suffer from limited specificity and methodological variability, and may be influenced by dietary factors and comorbidities [47,48]. In contrast, SH group quantification provides a direct readout of thiol availability and oxidative thiol modification, both of which are increasingly recognized as central to redox biology and redox-mediated signaling [49]. It is also plausible that the limited prognostic relevance of TBARS in our cohort reflects the early disease stage in the enrolled patients. In newly diagnosed individuals, the systemic antioxidant barrier, represented by circulating SH groups, may still be relatively preserved [36,37]. These thiol-based defenses can neutralize reactive species and thereby limit lipid peroxidation, attenuating the accumulation of measurable TBARS [50]. In this compensatory phase, TBARS concentrations might underestimate the underlying oxidative burden or its clinical impact [50]. This dynamic interplay between antioxidant availability and oxidative damage suggests that TBARS could gain prognostic significance at later stages, when thiol reserves are progressively depleted and redox imbalance becomes unopposed.

Prospective studies evaluating longitudinal trajectories of redox biomarkers over the IPF disease course are warranted to elucidate these temporal relationships. Importantly, our study population consisted of patients with newly diagnosed IPF, most of whom were receiving antifibrotic treatment. No significant differences in antifibrotic drug use were observed between survivors and non-survivors, suggesting that the prognostic value of SH concentrations is independent of pharmacologic modulation. This is clinically relevant, as it underscores the potential of SH groups as a biomarker applicable across different therapeutic contexts. Moreover, while lung function parameters such as %FVC and %TLC were associated with survival, SH concentrations retained prognostic significance even after adjustment for these variables, indicating additional prognostic value beyond conventional measures of pulmonary impairment.

The observed trend toward an association between lower SH concentrations and more advanced disease stages also warrants attention. Although this trend did not reach statistical significance, it suggests that thiol depletion may reflect disease burden or more aggressive phenotypes. Future studies should explore whether longitudinal changes in SH concentrations parallel clinical deterioration or predict acute exacerbations.

Notably, while established IPF biomarkers such as KL-6, MMP-7, and surfactant protein D primarily track alveolar epithelial damage and extracellular matrix remodeling, plasma SH concentrations reflect systemic redox imbalance and oxidative stress pathways [51,52]. From a clinical perspective, plasma SH offers key methodological and practical advantages over these traditional markers. Specifically, while the quantification of KL-6 or MMP-7 requires expensive and time-consuming commercial immunoassay kits, plasma SH can be measured using a highly cost-effective, rapid, and easily accessible ‘in-house’ spectrophotometric assay. This method provides high reproducibility and optimal analytical stability, characterized by low coefficients of variation (CVs). Consequently, plasma SH represents a promising, affordable, and complementary tool that could be integrated with tissue-remodeling markers to improve prognostic assessment in patients with IPF.

Our study has some limitations. First, with 38 events, we cannot completely exclude the risk of model overfitting in the multivariate analysis. However, we limited this risk by constructing two separate multivariate models each including a maximum of four predictors, resulting in an events-per-variable ratio of 9.5–12.7. In addition, multi-collinearity was formally assessed and was not a concern. The consistency of the results across both models supports the robustness of the independent association between plasma SH concentrations and mortality. Second, the single-center design and limited sample size may affect generalizability and statistical power, particularly for the analysis of TBARS. Furthermore, our study relied on a single baseline measurement of plasma SH and TBARS, precluding the evaluation of their longitudinal dynamics over time, and lacked an independent external cohort for validation.

We acknowledge that quantitative assessment of fibrosis extent on HRCT was not available for a sufficient number of patients and could not be included in the multivariate models. However, adjustment for multiple established physiological markers of disease severity (%FVC, %DLCO, %TLC, and GAP stage) was performed. Moreover, although plasma SH concentrations were normalized to total plasma protein content, specific serum albumin levels were not available in this study. Since albumin is the most abundant plasma protein and a major contributor to the circulating thiol pool, we cannot exclude the possibility that minor variations in albumin concentration may have slightly influenced PSH values. Additionally, while the study employed rigorous diagnostic criteria and standardized methodologies, the observational nature of the analysis precludes causal inference. It remains unclear whether thiol depletion represents a marker or a mediator of fibrotic progression. Future multicenter studies incorporating systematic quantitative HRCT analysis will be important to confirm whether plasma SH concentrations provide independent prognostic information beyond radiological fibrosis burden. Furthermore, due to the exploratory nature of this study and the relatively small sample size, we did not evaluate the incremental prognostic value of plasma SH concentrations over established clinical predictors. Consequently, while plasma SH concentrations represent a promising independent prognostic indicator in IPF, larger prospective studies are required to formally assess its clinical utility and performance in prognostic models.

## 5. Conclusions

In conclusion, our results indicate that reduced plasma SH concentrations are independently associated with increased all-cause mortality in patients with newly diagnosed IPF, suggesting that systemic thiol depletion may reflect a clinically relevant redox imbalance in this disease. Unlike TBARS, plasma SH concentrations showed a consistent association with survival after adjustment for established prognostic parameters, supporting their potential role as a complementary prognostic biomarker in IPF.

## Figures and Tables

**Table 1:** Baseline demographic, clinical, and laboratory characteristics of the study population.

	Global Cohort (*n* = 88)	Survivors (*n* = 50)	Non-Survivors (*n* = 38)	*p*-Value
Age, years	69.7 ± 7.4	69.7 ± 7.5	69.8 ± 7.4	0.95
Gender (M/F)	67/21	35/15	32/6	0.12
BMI (kg/m^2^)	27.6 ± 3.6	27.1 ± 3.3	28.2 ± 3.9	0.17
Smoking status, n (no/former/yes)	17/66/5	12/36/2	5/30/3	0.36
Diabetes, n (no/yes)	76/12	44/6	32/6	0.61
Arterial hypertension, n (no/yes)	50/38	30/20	20/18	0.49
Cerebrovascular diseases, n (no/yes)	82/6	46/4	36/2	0.62
Atrial arrhythmias, n (no/yes)	80/8	47/3	33/5	0.25
GERD, n (no/yes)	61/27	34/16	27/11	0.76
FEV_1_, (%)	84.8 ± 21.9	87.5 ± 23.6	81.3 ± 19.1	0.19
FVC, (%)	76.7 ± 20.1	80.4 ± 20.6	71.9 ± 18.6	0.047
FEV_1_/FVC, (%)	88.7 (84.9–92.9)	88.4 (84.5–94.1)	89.5 (85.9–92.6)	0.60
TLC, (%)	75.0 (65.0–86.7)	80.3 (68.1–88.8)	69.8 (57.1–81.4)	0.022
DLCO, (%)	52.5 (37.6–65.8)	60.1 (39.3–68.1)	48.1 (35.1–57.0)	0.11
6MWT, (meters)	343 ± 162	372 ± 160	302 ± 158	0.046
GAP stage, (I/II/III)	42/37/9	29/16/5	13/21/4	0.07
Therapy, (no/Pirfenidone/Nintedanib)	4/44/40	1/25/24	3/19/16	0.40
Follow-up duration, (months)	51.9 ± 27.5	61.8 ± 25.9	38.8 ± 24.2	˂0.001

6MWT: six-minute walk test; BMI: body mass index; DLCO: diffusion capacity for carbon monoxide; F: Females; FEV_1_: forced expiratory volume in the 1st second; FVC forced vital capacity; GAP: gender–age–physiology score; GERD: gastroesophageal reflux disease; M: males; TLC total lung capacity; Therapy status refers to antifibrotic treatment during follow-up; at baseline, all patients were treatment-naïve.

**Table 2:** Univariate Cox regression analysis showing hazard ratios for the studied variables.

	HR	95% CI	*p*-Value
Age	0.9942	0.9528 to 1.0374	0.7887
Gender (M/F)	0.4478	0.1857 to 1.0800	0.0737
BMI	1.0670	0.9813 to 1.1602	0.1287
Smoking status (no/former/yes)	1.7993	0.9090 to 3.5616	0.0918
Diabetes (no/yes)	1.8938	0.7815 to 4.5890	0.1573
Arterial hypertension (no/yes)	1.2281	0.6483 to 2.3264	0.5285
Cerebrovascular diseases (no/yes)	0.8202	0.1965 to 3.4232	0.7857
Atrial arrhythmias (no/yes)	1.4306	0.5570 to 3.6747	0.4569
GERD (no/yes)	0.8790	0.4355 to 1.7743	0.7190
FEV_1_, (%)	0.9920	0.9775 to 1.0067	0.2855
FVC, (%)	0.9803	0.9630 to 0.9978	0.0276
FEV_1_/FVC, (%)	1.0001	0.9999 to 1.0003	0.4179
TLC, (%)	0.9750	0.9578 to 0.9926	0.0054
DLCO, (%)	0.9807	0.9623 to 0.9995	0.045
6MWT	0.9984	0.9964 to 1.0005	0.1340
GAP stage (I/II/III)	1.6369	1.0095 to 2.6541	0.0457
Therapy (no/Pirfenidone/Nintedanib)	0.8140	0.4345 to 1.5251	0.5206
PSH	0.6567	0.4915 to 0.8775	0.0045
TBARS	1.1737	0.9855 to 1.3979	0.0725

6MWT: six-minute walk test; BMI: body mass index; DLCO: diffusion capacity for carbon monoxide; FEV_1_: forced expiratory volume in the 1st second; FVC: forced vital capacity; GAP: gender–age–physiology score; GERD: gastroesophageal reflux disease; PSH: Plasma SH; TBARS: thiobarbituric acid-reactive substance; TLC: total lung capacity.

**Table 3:** Multivariate Cox regression models for the studied variables.

	HR	95% CI	*p*-Value
**Model 1**			
Stage	--	--	--
TLC (%)	0.9770	0.9567 to 0.9976	0.0291
PSH	0.6637	0.4979 to 0.8847	0.0052
**Model 2**			
DLCO (%)	0.9754	0.9536 to 0.9977	0.0311
FVC (%)	--	--	--
TLC (%)	--	--	--
PSH	0.6063	0.4427 to 0.8302	0.0018

CI: confidence interval; DLCO: Diffusion capacity for carbon monoxide; FVC: forced vital capacity; HR: hazard ratio; PSH: plasma SH; TLC: total lung capacity. Two separate models were built to avoid multicollinearity between GAP stage and its components (%FVC and %DLCO).

## Data Availability

The original contributions presented in this study are included in the article. Further inquiries can be directed to the corresponding author.
